# Continuing Professional Development‐Medical Imaging

**DOI:** 10.1002/jmrs.679

**Published:** 2023-05-12

Maximise your CPD by reading the following selected article and answer the five questions. Please remember to self‐claim your CPD and retain your supporting evidence. Answers will be available via the QR code and online at www.asmirt.org/news-and-publications/jmrs, as well as published in JMRS – Volume 70, Issue 4 December 2023.

## Medical Imaging – Original Article

### Digital radiography reject analysis: A comparison between two radiology departments in New Zealand

Bantas G, Sweeney JR, Mdletshe S. (2023), *J Med Radiat Sci*. https://doi.org/10.1002/jmrs.654

1. Why is analysis of image rejection important?
  - It is a valuable tool for x‐ray equipment quality assurance and dose output calibrations.
  - It is a requirement of the National Safety and Quality Health Service (NSQHS) Standards.
  - It forms part of a quality assurance process and provides a measure of efficiency of radiology departments.
  - It is not considered important as it is quick and easy to retake an image with digital radiography.
2. For direct digital radiography, what image rejection rate is recommended by the American Association of Physicists in Medicine (AAPM)?
  - 2% ±0.5%
  - 5% ±1%
  - 8% ±2%
  - 15% ±3%
3. In this study, what was the most common reason for image rejection?
  - Positioning errors
  - Collimation errors
  - Patient movement
  - Incorrect exposure factors
4. In this study, which anatomic region had the greatest image rejection rate?
  - Head
  - Spine
  - Pelvis/Hip
  - Shoulder
5. Which of the following was identified as a limitation of this study?
  - The sample size was too small to produce reliable results, more images are required to increase the statistical power.
  - Different versions of the image reject analysis software were installed in the different radiography systems, resulting in a lack of standardised image rejection reasons.
  - The medical imaging technologists involved in this study received formal training in image quality assessment, hence results should not be generalised to the whole profession.
  - All acquired images in this study were from outpatient examinations only, hence a more diverse range of images would provide a more accurate image rejection rate.

### Recommended further reading:

1. Atkinson S, Neep M, Starkey D. Reject rate analysis in digital radiography: an Australian emergency imaging department case study. J Med Radiat Sci. 2020 Mar;67(1):72‐79. doi: 10.1002/jmrs.343
2. Stephenson‐Smith B, Neep MJ, Rowntree P. Digital radiography reject analysis of examinations with multiple rejects: an Australian emergency imaging department clinical audit. J Med Radiat Sci. 2021 Sep;68(3):245‐252. doi: 10.1002/jmrs.468
3. Hofmann B, Rosanowsky TB, Jensen C, Wah KH. Image rejects in general direct digital radiography. Acta Radiol Open. 2015 Oct 8;4(10):2058460115604339. doi: 10.1177/2058460115604339
4. Jones AK, Heintz P, Geiser W, Goldman L, Jerjian K, Martin M, Peck D, Pfeiffer D, Ranger N, Yorkston J. Ongoing quality control in digital radiography: Report of AAPM Imaging Physics Committee Task Group 151. Med Phys. 2015 Nov;42(11):6658‐70. doi: 10.1118/1.4932623

### Answers


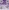


Scan this QR code to find the answers, or visit www.asmirt.org/news-and-publications/jmrs

